# Spectrophotometry assays to determine G6PD activity from Trinity Biotech and Pointe Scientific G6PD show good correlation

**DOI:** 10.1186/s13104-018-3964-7

**Published:** 2018-12-04

**Authors:** Mohammad Shafiul Alam, Mohammad Golam Kibria, Nusrat Jahan, Ric N. Price, Benedikt Ley

**Affiliations:** 1 Infectious Diseases Division, International Centre for Diarrheal Diseases Research, Bangladesh, Mohakhali, Dhaka, Bangladesh; 2 Global and Tropical Health Division, Menzies School of Health Research and Charles Darwin University, Darwin, Australia; 3 Centre for Tropical Medicine and Global Health, Nuffield Department of Clinical Medicine, University of Oxford, Oxford, UK

**Keywords:** G6PD, Malaria, Spectrophotometry

## Abstract

**Objectives:**

Spectrophotometry kits from Pointe Scientific (PS; USA) were compared to kits from Trinity Biotech (Trinity; Ireland) in 50 venous blood samples from purposively selected individuals in Bangladesh. Repeatability and inter-assay variability were assessed by Students t-test, Bland-Altman plot and Pearson correlation coefficient (r). The median glucose-6-phosphate dehydrogenase (G6PD) activity of all G6PD normal participants was calculated per assay and defined as 100% activity. Performance was calculated considering 30% and 70% cut off activities and Trinity as reference.

**Results:**

The intra-assay correlation of Trinity (r = 0.9841, p < 0.001) and PS (r = 0.9833, p < 0.001) did not differ significantly (p = 0.904). Both assays were closely correlated (r = 0.9799, p < 0.001), with a mean difference of 0.1 U/gHb (95% limit of agreement: − 1.32 to 1.57). At 30% cut off PS had a sensitivity of 100% (95% confidence interval (95 CI) 59.0–100.0) and specificity of 100% (95% CI 91.8 to 100.0), at 70% cut-off of 100% (95% CI 79.4–100.0) and 97.1% (95% CI 84.7–99.9) respectively. The G6PD assay from PS is a reliable alternative to the assay from Trinity.

**Electronic supplementary material:**

The online version of this article (10.1186/s13104-018-3964-7) contains supplementary material, which is available to authorized users.

## Introduction

Glucose-6-phosphate dehydrogenase (G6PD) is the rate limiting step of the pentose-phosphate-pathway (PPP), which reduces nicotinamide adenine dinucleotide phosphate (NADP^+^) to nicotinamide adenine dinucleotide phosphate hydrogen (NADPH) [1]. G6PD deficiency is the most common enzymopathy worldwide, affecting approximately 400 million people globally [2]. G6PD deficiency does not affect life expectancy in most cases it is however a known risk factor for hyperbilirubinemia and kernicterus [3] and can result in hemolysis induced by specific compounds such as fava beans, sulphonamides, quinolones, dapsone and the 8-aminoquinoline class of antimalarials [2].

The reference method for the quantification of G6PD enzyme activity is spectrophotometry [4–6], a method based on the colorimetric detection of NADPH. In brief a defined amount of hemolysate is added to a solution containing glucose-6-phosphate and NADP, which results in the formation of NADPH facilitated through G6PD. Depending on the activity of the G6PD, NADPH production varies and can be measured at a wavelength of 340 nm over a predefined time interval at standardized temperatures [7]. There are a number of spectrophotometry kits from different providers available, one of the most common is that manufactured by Trinity Biotech (Trinity; Ireland). In 2017 the company suspended their production due to difficulties in maintaining quality standards for their reagents. Pointe Scientific (PS; USA) also produce reagents for G6PD spectrophotometry that are similar to Trinity in their operational characteristics, however are designed for a higher optimal measuring temperature. The aim of this study was to compare the intra and inter assay variability derived from spectrophotometry using reagents from Trinity and PS.

## Main text

### Methods

Venous blood (total 3 mL) was collected from purposively selected participants with known G6PD activity in the Chittagong Hill Tracts, Bangladesh to cover the broadest possible range of G6PD activities. Samples were stored at 4 °C and G6PD activity measured within 24 h by spectrophotometry in a reference laboratory in Dhaka.

Haemoglobin (Hb) was measured by a complete blood count (CBC) using a XN-1000 (Sysmex Corporation, Kobe, Japan) immediately before spectrophotometry; samples were tested no more than an hour apart on a Shimadzu UV-1800 (Shimadzu, Japan) with kits from Trinity (Cat. No.: 345-B) and PS (Cat. No.: G7583) according to manufacturer instructions. Each kit was run in duplicate, with a measuring temperature of 30 °C for Trinity and 37 °C for PS, all measurements were done by the same two experienced and well-trained laboratory technicians who had a Master degree in a relevant field. G6PD activity was calculated from the change in absorbance at 340 nm over a period of 5 min, as per the manufacturer’s instructions, and the derived G6PD activity (U/dL) was normalized by Hb (U/gHb). G6PD deficient (Cat. No.: HC-108DE), intermediate (Cat. No.: HC-108IN) and normal controls (Cat. No.: HCS-108) (all from ACS Inc., Fishers, USA) were run daily prior to sample testing.

Repeatability was assessed per assay by correlating the first and second measurement of each kit. For the inter-assay comparison, the mean of both normalized results was calculated and compared by Students t-test, Bland Altman plots and the Pearson correlation coefficient (r).

100% G6PD activity was defined for each assay by calculating the median activity of all participants that had been found to have G6PD activity above 70% of the adjusted male median (AMM) in earlier studies [4, 8]. Each individual was then categorized as having below 10%, below 30% and below 70% activity. The proportions within each category were compared between kits using the McNemars test for correlated proportions. The sensitivity and specificity of PS were calculated at each threshold assuming Trinity as the gold standard assay [4].

### Results

A total of 50 samples were analysed by both kits and no results were excluded from the analysis. The repeatability of Trinity (r = 0.9841, p < 0.001) and PS (r = 0.9833, p < 0.001) was very high and did not differ significantly among the assays (p = 0.904).

**Fig. 1:**
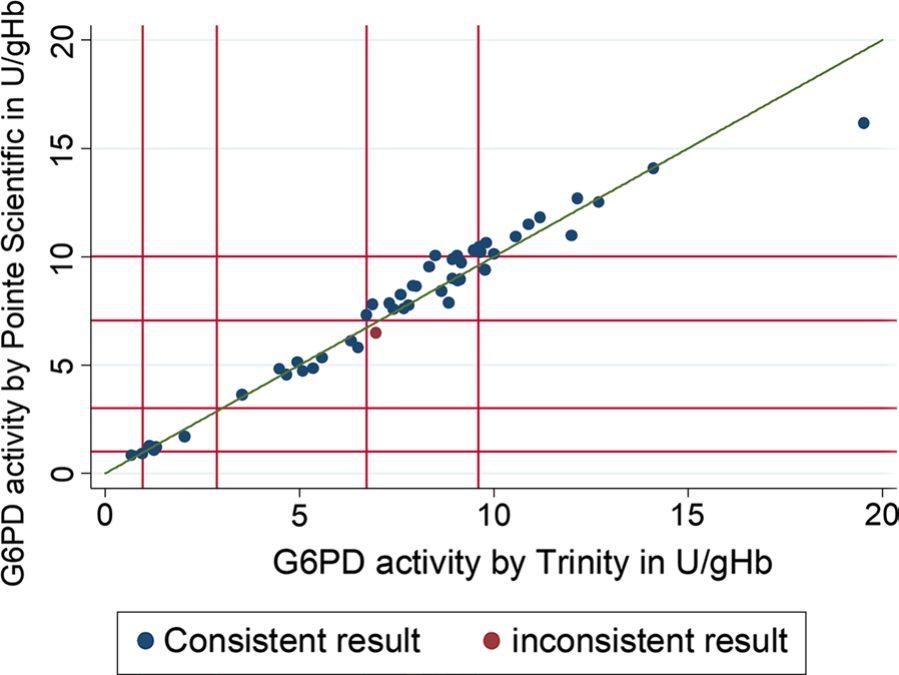
Scatter plot mean Trinity Biotech (Trinity) results vs. mean Pointe Scientific (PS) results. Red lines from origin outwards indicate 10%, 30%, 70% and 100% cut off activity

The results from PS correlated closely with results from Trinity (r = 0.9799, p < 0.001; Fig. 1). The mean of the activities derived by Trinity was 7.6 U/gHb (95% confidence interval (95% CI) 6.53–8.64) compared to 7.7 U/gHb (95% CI 6.67–8.75) derived by PS; p = 0.126), with 95% limits of agreement ranging from − 1.32 to 1.57 U/gHb.

100% G6PD activity was based on 24 samples and was 10.1 U/gHb (interquartile range (IQR): 8.98–11.24) for PS and 9.6 U/gHb (IQR: 8.88–11.04) for Trinity. When results were categorized, 1 discrepant result (2%) was observed (Table 1 and Fig. 1), and the proportion of samples classified as being below 30% and 70% did not differ significantly (p = 1.000 and p = 0.317 respectively). At the 30% threshold the sensitivity of PS was 100% (95% CI 59.0–100.0) with a specificity of 100% (95% CI 91.8 to 100.0), at the 70% threshold the corresponding values were 100% (95% CI 79.4–100.0) and 97.1% (95% CI 84.7–99.9) respectively.

**Table 1:** Distribution of categorized result / test kits

		Trinity				
*Pointe Scientific*		< 10%^a^	10% to < 30%^a^	30% to < 70%^a^	> 70%^a^	Total
	< 10%^b^	2	0	0	0	2
	10% to < 30%^b^	1	5	0	0	5
	30% to < 70%^b^	0	0	9	1	10
	> 70%^b^	0	0	0	33	33
	Total	2	5	9	34	50

^a^ 100% G6PD activity Trinity: 9.6 U/gHb

^b^ 100% G6PD activity Pointe Scientific: 10.1 U/gHb

### Discussion

These findings demonstrate that PS is a reliable alternative to the assays from Trinity for the quantification of G6PD enzyme activity by spectrophotometry. Intra and inter assay correlation for the assays was excellent, suggesting that a single measurement would be sufficient providing good quality control and well-informed technicians. No sample with G6PD activity below the clinically relevant 30% cut off activity was misclassified, one sample was considered below 70% by PS but not by Trinity [9], however the respective sample was borderline (Fig. 1). Both assays showed a very small mean difference, suggesting that population specific cut offs [4] established earlier by Trinity kits are likely to be applicable to results obtained using the PS kits, although further confirmatory studies are warranted.

The operational characteristics of both assays are similar, with the exception that PS requires a higher temperature if no temperature correction factor is to be applied. This minor modification can be achieved on most if not all spectrophotometers suitable for Trinity kits. The cost of the Trinity kit in 2016 was 3.60 USD/test, whereas the cost of each PS kit is currently approximately 2.00 USD/test (these prices refer to the Australian market and are likely to differ depending on location and distributor).

### Conclusion

These results demonstrate that both assays work well if performed by well trained technicians and when maintaining quality control measures. Given that assays from Trinity are no longer available, assays from PS provide a good alternative at lower costs.

## Limitations

All samples were tested on a high—end machine, assays may perform at lower accuracy on different machines. Inter-reader variability was not assessed in the course of this study, however given the consistent results, good performance observed and extensive experiences of both laboratory technicians we do not believe this has impacted on our findings.

## Additional file

**Additional file 1.** Database with all relevant information

## Abbreviations

G6PD: glucose-6-phosphate dehydrogenase
PPP: pentose-phosphate-pathway
NADP^+^: nicotinamide adenine dinucleotide phosphate
NADPH: nicotinamide adenine dinucleotide phosphate hydrogen
Trinity: Trinity Biotech (Ireland)
PS: Pointe Scientific (USA)
Hb: haemoglobin
CBC: complete blood count
USD: United States Dollar
